# Progress on innate immune evasion and live attenuated vaccine of pseudorabies virus

**DOI:** 10.3389/fmicb.2023.1138016

**Published:** 2023-03-03

**Authors:** Zhenyu Nie, Shunfan Zhu, Li Wu, Ruolin Sun, Jianhong Shu, Yulong He, Huapeng Feng

**Affiliations:** ^1^Department of Biopharmacy, College of Life Sciences and Medicine, Zhejiang Sci-Tech University, Hangzhou, China; ^2^Shaoxing Academy of Biomedicine, Zhejiang Sci-Tech University, Shaoxing, China; ^3^Department of Biology, College of Life Sciences, China Jiliang University, Hangzhou, China

**Keywords:** pseudorabies virus, innate immunity, immune evasion, live attenuated vaccine, recombinant vaccine

## Abstract

Pseudorabies virus (PRV) is a highly infectious disease that can infect most mammals, with pigs as the only natural host, has caused considerable economic losses to the pig husbandry of the world. Innate immunity is the first defense line of the host against the attack of pathogens and is essential for the proper establishment of adaptive immunity. The host uses the innate immune response to against the invasion of PRV; however PRV makes use of various strategies to inhibit the innate immunity to promote the virus replication. Currently, live attenuated vaccine is used to prevent pig from infection with the PRV worldwide, such as Bartha K61. However, a growing number of data indicates that these vaccines do not provide complete protection against new PRV variants that have emerged since late 2011. Here we summarized the interactions between PRV and host innate immunity and the current status of live attenuated PRV vaccines to promote the development of novel and more effective PRV vaccines.

## 1. Introduction

PRV is the pathogen that causes pseudorabies (PR), also known as Aujeszky’s disease (AD; Zhou et al., 2019). PRV belongs to the alpha-herpesvirus subfamily with a broad host range, and although pigs are its natural host, many mammals, including humans, cats, dogs, goats, and cattle, can also be infected (Wong et al., 2019; Laval and Enquist, 2020; Li et al., 2020). PRV infection in pigs can cause respiratory, reproductive, and neurological symptoms (Lu J. J. et al., 2021). Pig at different stages of growth present different symptoms. PRV-infected newborn piglets show neurological symptoms with mortality up to 100% (Zheng et al., 2022), while adult pigs generally show only mild respiratory symptoms, but can establish a lifelong latent infection in their nervous system that can be reactivated and become a dynamic reservoir of the virus (Lu J. J. et al., 2021; Chen J. et al., 2022).

PRV possesses a double-stranded DNA genome of approximately 143 kb size, tightly arranged with at least 70 open reading frames, and containing two inverted repeats (Klupp et al., 2004). Several of PRV proteins exert the function of modulating the host’s innate immune responses, mainly including the interferon (IFN) system, apoptosis, and autophagy, to facilitate viral production. Innate immunity responds rapidly to viral infections, but pathogen clearance and prevention of reinfection rely critically on adaptive immunity, and a high-quality innate immune response is a prerequisite for establishing adaptive immunity (Le Bon et al., 2001; Iwasaki and Medzhitov, 2015; Castro Dopico et al., 2022). Therefore, understanding the innate immune evasion mechanism of PRV is important for vaccine development.

Since the PRV epidemic, the live attenuated PRV vaccines, representative of Bartha K61, play a critical role in preventing and controlling this disease. However, new variants have been raised since 2011 in China. Bartha K61 could not provide complete protection from PRV variant infection (Yu et al., 2014; Tong et al., 2015). New vaccine development is a requirement, and some scholars believe that enhanced innate immunity to cause viral attenuation (Tulloch et al., 2014). Breakdown of the innate immune evasion of the virus can reduce the virulence of the virus while establishing a robust immune response (Deng et al., 2017, 2020). We summarized the multiple strategies of innate immune evasion of PRV, mainly including the IFN system, autophagy, and apoptosis, and current progress on PRV live attenuated vaccines. We expect that this review could provide some ideas to develop vaccines against novel PRV variants.

## 2. Innate immune regulation mechanisms of PRV

Viral infection induces innate immune responses and activates an antiviral innate immune response through pattern recognition receptor (PRR) recognition of pathogen-associated molecular patterns (PAMPs; Akira et al., 2006; Takeuchi and Akira, 2010). It has been reported that the host can recognize PRV infection by several PRRs: Toll-like receptor 2 (TLR2), Cyclic guanosine monophosphate-adenosine monophosphate synthase (cGAS), RIG-I-like receptors (RLRs), DEAD (Asp-Glu-Ala-Asp) box polypeptide 41 (DDX41), and DNA-dependent activator of IFN-regulatory factors (DAI; Xie et al., 2010; Zhu et al., 2014; Wang J. et al., 2015; Laval et al., 2019; Li et al., 2021; Liu et al., 2021; Zhao N. et al., 2022). Subsequently, specific signaling pathways are activated to produce interferons, pro-inflammatory factors, and chemokines in response to pathogen infection (Akira et al., 2006; Rehwinkel and Gack, 2020).

**Figure 1:**
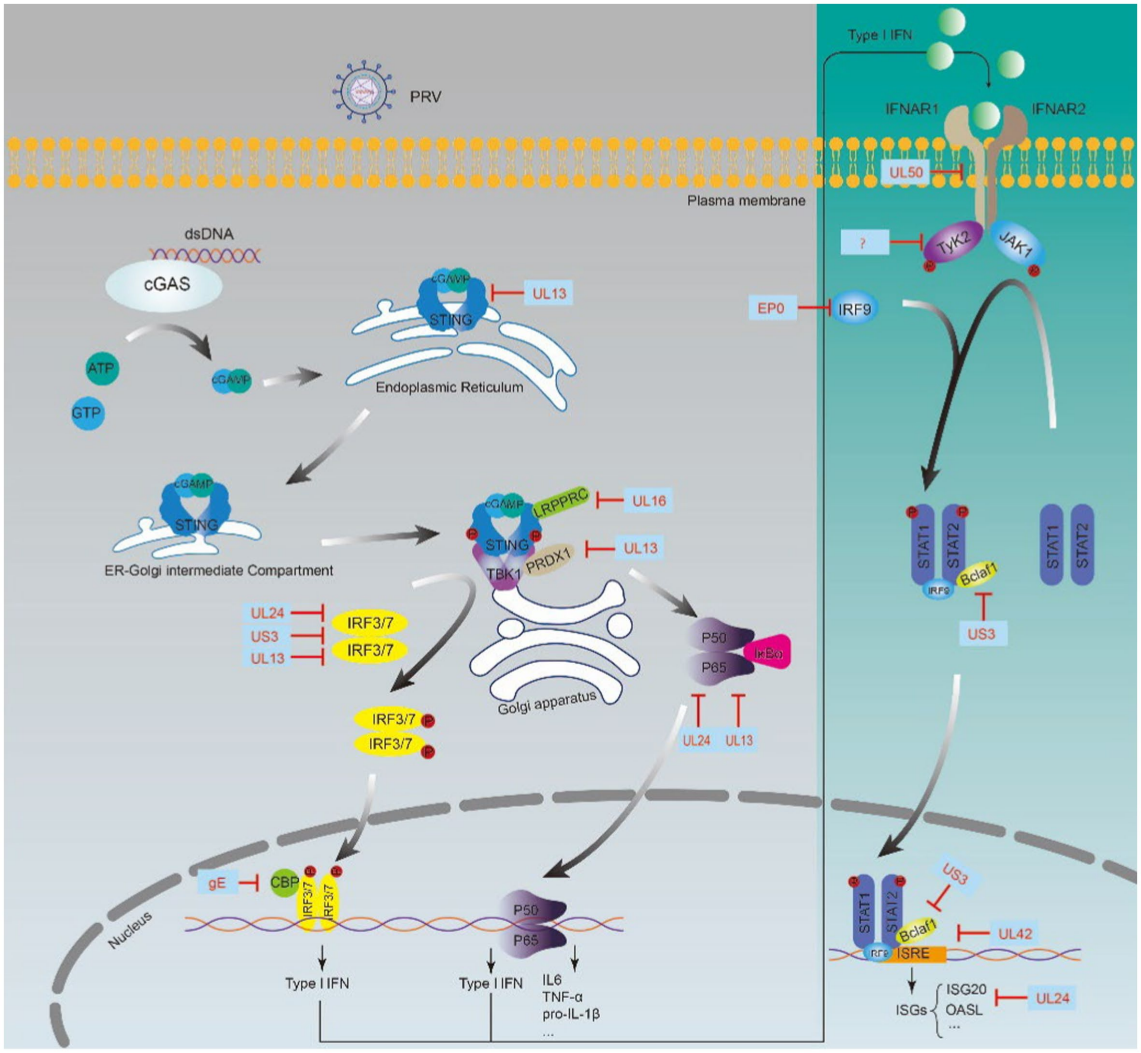
PRV regulates IFN-mediated innate immune responses by encoding multiple proteins.

We summarized the PRV-encoded proteins involved in the regulation of the innate immune system, focusing on those involved in escaping IFN-mediated immune responses (Figure 1; Table 1).

**Table 1:** PRV factors interact with the host’s innate immune system.

PRV Factors	Target Pathway	Molecular mechanism	References
gE	IFN production	Degrading CBP to interrupt the enhanced assembly of IRF3 and CBP	Lu M. et al. (2021)
	Apoptosis	Activation of ERK 1/2 signaling and consequently degradation of the pro-apoptotic protein Bim	Pontes et al. (2016)
UL24	IFN production	Degradation of IRF7 *via* the proteasome pathway	Liu et al. (2021)
	IFN signaling pathway	Inhibiting the transcription of OASL	Chen et al. (2021a)
		Inhibiting the transcription of ISG20	Chen et al. (2021b)
	NF-κB pathway	Degradation of p65 *via* the proteasome pathway	Wang T. -Y. et al. (2020)
US3	IFN production	Interacting with IRF3, blocking IRF3 activation, and degrading IRF3 *via* the proteasome pathway	Xie et al. (2021)
	IFN signaling pathway	Degradation of Bclaf1 *via* the proteasome pathway	Qin et al. (2019)
	Apoptosis	Activation of PI3-K/Akt and NF-κB pathways	Chang et al. (2013)
	Autophagy	Activation of PI3-K/Akt pathway	Sun et al. (2017)
UL13	IFN production	Degradation of PRDX1 *via* the proteasome pathway	Lv et al. (2021)
		Targeting IRF3 for ubiquitination and degradation	Lv et al. (2020)
		Phosphorylation of IRF3	Bo et al. (2020)
		Regulation of NF-κB activation inhibits the transcription of RIG-I and MDA5	Zhao N. et al. (2022)
		Accelerated ubiquitination of STING in cooperation with E3 ligase RNF5	Kong et al. (2022)
	DNA damage response	Interacting with H2AX and phosphorylating H2AX	Ming et al. (2022a)
UL16	NF-κB pathway	Interacting with LRPPRC and attenuates its activation of NF-κB	Xu et al. (2022a)
UL42	IFN signaling pathway	Blocking the association of ISGF3 and ISRE	Zhang et al. (2021)
UL50	IFN signaling pathway	Promoting lysosomal degradation of IFNAR1	Zhang et al. (2017)
EP0	IFN signaling pathway	Inhibition of IRF9 transcription	Wang M. et al. (2022)

### 2.1. IFN-I system

The IFN-I-mediated innate immune response is one of the most effective defense strategies of the host against viral infection. However, viruses have developed various strategies to evade host immunity during their long evolution (Garcia-Sastre, 2017; O’Connor and Sen, 2021; Verzosa et al., 2021; Ye et al., 2022). PRV regulates the host innate immune responses in several strategies. The expression of a subset of genes commonly induced by IFN-β was decreased when the cells were infected with wild-type PRV strains using microarray technology (Brukman and Enquist, 2006b). Upon viral infection, viral DNA is firstly recognized by cGAS, a cytoplasmic DNA sensor, resulting in a conformational switch in cGAS to catalyze the production of the second messenger cGAMP, then cGAMP binds to STING located on the endoplasmic reticulum. They translocate to the Golgi apparatus to recruit TANK-binding kinase 1 (TBK1) and IRF3/7 and phosphorylate IRF3/7, and finally phosphorylated IRF3/7 is transported into the nucleus and activates transcription of the genes encoding IFN-I (Chen et al., 2016; Motwani et al., 2019). Subsequently, the JAK–STAT pathway is activated by IFN-I, and initiates the transcription of hundreds of ISGs, which could regulate cytokines (Brukman and Enquist, 2006b). PRV encodes a series of proteins to circumvent IFN-I-mediated innate immunity, with the primary targets cGAS-STING signaling pathway and IFN-I signaling pathway (Table 1; Zhang and Tang, 2021).

STING is one of the critical proteins in the cGAS-STING pathway, and a robust study showed that PRV UL13 recruits the E3 ligase RING-finger protein 5 (RNF5) to promote the K27-/K29-linked ubiquitination and degradation of STING (Kong et al., 2022). Peroxiredoxin 1 (PRDX1) contributes to IFN induction by interacting with TBK1 and IκB kinase ε (IKKε), and Lv et al. demonstrated that UL13 degrades PRDX1 to inhibit IFN production depending on its kinase activity (Lv et al., 2021). UL13 has been shown to degrade IRF3 through ubiquitination and proteasome-mediated degradation (Lv et al., 2020). Bo et al. found that UL13 restricted IFN-β transcription by screening 50 PRV proteins. Further study found that UL13 used its kinase activity to induce IRF3 phosphorylation and thus attenuate the binding of IRF3 to its promoter (Bo et al., 2020). IRF3 activity was also inhibited by gE and US3 in multiple ways. gE inhibits IFN production by degrading CREB-binding protein (CBP), a partner of IRF3 (Lu M. et al., 2021). US3 inhibits the activity of IRF3, mechanistically degrading IRF3 *via* the proteasome pathway (Xie et al., 2021). UL24 impairs the DNA sensing pathway by degrading IRF7 (Liu et al., 2021).

The JAK/STAT signaling pathway is important in IFN-I-mediated ISGs production. The JAK/STAT pathway was regulated extensively by PRV. PRV blocked the phosphorylation of STAT1, a vital node of the JAK/STAT signaling pathway (Brukman and Enquist, 2006b). Several viral proteins play an active role in this process, including EP0, US3, UL24, UL42, and UL50. The EP0 protein is required for antagonizing the natural host interferon response by repressing the transcription of IRF9 (Brukman and Enquist, 2006a; Wang M. et al., 2022). Bcl-2 associated transcription factor 1 (Bclaf1) maintains STAT1 and STAT2 phosphorylation and promotes ISGF3 binding to ISRE by directly interacting with ISRE and STAT2 to transcribe ISG (Qin et al., 2019). US3 inhibits the production of ISGs by degrading Bclaf1 through the proteasome pathway to facilitate the immune evasion of PRV (Qin et al., 2019). In addition, UL42 also interferes with the binding of ISGF3 to ISRE, and some other studies found that UL24 represses the transcription of ISGs, including OASL, ISG20, and so on (Zhang et al., 2021; Chen et al., 2021a,b).UL50 was found to degrade the IFN receptor to down-regulate the IFN-α immune response (Zhang et al., 2017). Janus kinase phosphorylates and conducts the antiviral signal of IFN, and recently it was found that PRV degrades Janus kinase by inducing proteasomal degradation (Yin et al., 2021). However, the mechanism involved is still unclear.

Nuclear factor-κB (NF-κB) has a vital role in regulating immunity, inflammatory response, cell proliferation, and survival (Li and Verma, 2002; Chen et al., 2014). The main form of NF-κB is a heterodimer composed of p50 and RelA subunits, which bind to IκB family proteins with a state of inhibited activity (Ghosh et al., 1998; Romero et al., 2020). When cells are subjected to various stimuli, including stress, cytokines, or invasion by pathogens, the NF-κB inhibitory protein IκBα is activated and separated from NF-κB and degraded *via* the proteasome pathway, and NF-κB is further translocated to the nucleus to activate transcription of various genes (Karin and Ben-Neriah, 2000; Santoro et al., 2003; Chang et al., 2013). The cellular NF-κB signaling pathway is profoundly affected by PRV infection. Recent studies have revealed that the PRV-encoded protein UL13 regulates NF-κB activation by inhibiting the transcription of RIG-I and MDA5, thereby inhibiting RLR-mediated IFN-β production from evading innate host immunity (Zhao N. et al., 2022). In addition, UL24 interacts with p65 and mediates p65 degradation of the proteasome pathway and thus impairs NF-κB activation (Wang T. -Y. et al., 2020).

In conclusion, PRV regulates the function of IFN from multiple aspects including IFN production and the IFN signaling pathway. There was a strong correlation between the ability of the virus to inhibit IFN signaling and virulence. The measles virus is attenuated by deleting STAT1/2 antagonists (Devaux et al., 2008). A critical study found that the glycoprotein gE/gI complex of PRV significantly inhibited IFN secretion by plasmacytoid dendritic cells (pDCs). The Bartha K61 attenuated vaccine strain with deleted gE/gI triggered a more robust IFN-I response *via* pDC than the wild-type (WT) PRV strain (Lamote et al., 2017). Restoration or even improvement of the IFN system damaged in wild-type PRV infection is a promising antiviral strategy. Although the IFN system is not the only determinant of viral virulence, it is still substantial. The critical role played by the immune escape factor of PRV in viral infections provides a potential target for vaccine and antiviral drug development.

### 2.2. Apoptosis, autophagy, and necroptosis

Apoptosis is a programmed cell death characterized by blistering of the cell membrane, chromatin condensation, and DNA fragmentation while maintaining membrane integrity to form apoptotic vesicles (Arandjelovic and Ravichandran, 2015). Apoptosis is a means of host resistance to pathogen invasion, causing premature death of infected cells resulting in abortion of viral proliferation and induction of specific immunity by mildly releasing antigens (Koyama et al., 2000; Zhu et al., 2021). PRV induced-apoptosis has been reported (Cheung et al., 2000; Aleman et al., 2001). The US3 and gE proteins of PRV not only antagonize the IFN system, but also inhibit apoptosis through different pathways. It was demonstrated that US3 activates PI3K/Akt and NF-κB pathways to inhibit apoptosis, and gE phosphorylates extracellular signal regulated kinase 1/2 (ERK1/2) to degrade the pro-apoptotic protein Bim (Chang et al., 2013; Pontes et al., 2016). The DNA damage response (DDR) is essential for maintaining genomic integrity, and leads to DNA repair, cell cycle arrest, and apoptosis depending on the degree of damage (Ming et al., 2022b). PRV infection causes DNA damage in host cells, leading to apoptosis, and recent research indicates a more in-depth dialogue between PRV and DDR (Lai et al., 2019). Phosphorylated H2AX is a hallmark of DDR, while UL13 of PRV can interact with H2AX and phosphorylate H2AX to facilitate its replication (Ming et al., 2022a). The leucine-rich PPR motif protein (LRPPRC) is a multifunctional protein, and the first apoptotic protein identified independently of caspase proteins. Recently, Xu et al. found that the UL16 protein of PRV interacted with LRPPRC and attenuated the activation of NF-κB by LRPPRC, which is a host restriction factor for PRV replication (Xu et al., 2022a). These genes are promising targets for PRV attenuation for the development of novel live attenuated vaccines.

Autophagy is used to renovate intracellular components and provide cells with vital energy and substances during cell starvation (Lum et al., 2005). An increasing number of studies show that autophagy is an integral part of the immune response (Levine and Deretic, 2007; Deretic, 2011; Xu et al., 2020). PRV can induce cellular autophagy to promote replication *via* the classical Beclin-1-Atg7-Atg5 pathway (Xu et al., 2018). The Wnt/β-Catenin signaling pathway regulates cell proliferation, differentiation, and survival (Mo et al., 2013). Recent studies have found that PRV infection significantly activates the Wnt/β-Catenin signaling pathway, which may promote viral proliferation by upregulating virus-induced autophagy (Wang C. et al., 2022). The autophagic pathway appears to be exploited by PRV, and Sun et al. showed that the exquisite regulation of autophagy by PRV, inducing cellular autophagy in the early stages of infection, which is independent of viral replication, and decreasing autophagy levels as replication progress. Further study revealed that US3 activates the PI3-K/AKT pathway to inhibit autophagy (Sun et al., 2017). The inhibition of cellular autophagy by US3 favors cell survival and may facilitate the replication of PRV. Bcl2-associated athanogene 3 (BAG3) is involved in regulating apoptosis, development, cytoskeleton organization, and autophagy (Rosati et al., 2011). BAG3 inhibits PRV replication, and the PRV protein UL56 interacts with BAG3 and degrades BAG3 *via* the lysosomal pathway; however, this phenomenon cannot be observed in WT PRV infection (Lyu et al., 2020). Some other protein of PRV probably antagonizes the activity of UL56 degradation to BAG3, and the details still need to be further explored and BAG3 could be as a potential target for generation of stable cell lines for high level production of live attenuated PRV vaccine.

Necroptosis is a form of cell death distinct from apoptosis (Vandenabeele et al., 2010; Wang H. et al., 2014). Activation of the mixed-spectrum kinase structural domain (MLKL) is manipulated with the help of receptor-interacting protein kinase 3 (RIPK3) and its chaperones (RIPK1, TRIF, DAI or others), leading to cell rupture to release a large number of damage-associated molecules and triggering an inflammatory response (Kaczmarek et al., 2013; Pasparakis and Vandenabeele, 2015; Zhang G. et al., 2022). PRV causes cell necroptosis in a RIPK3-dependent manner, and inhibition of necroptosis promotes PRV replication (Gou et al., 2021). Whether PRV blocks necrosis is unclear, but some other herpesviruses such as mouse cytomegalovirus (MCMV) encodes M45/vIRA to block RIPK3 activity, and similarly, HSV-1 encodes a ribonucleotide reductase large subunit (ICP6) that interacts with RIPK3 in human cells to inhibit necroptosis (Upton et al., 2012; Guo et al., 2015). These information indicates PRV probably has some potential ability to prohibit the cell necroptosis to facilitate its replication.

Apoptosis, autophagy, and necrosis are the three classical forms of cell death, and over time, the independence and relevance of these forms of cell death have been increasingly recognized (Chen et al., 2018). Multiple types of cell death can be induced simultaneously when cells are stimulated and can interconvert (Leist and Jäättelä, 2001). The relationship between PRV infection and host mortality is complex, and underlying the interaction between PRV and host is essential for developing novel vaccines and antiviral drugs against PRV infection.

## 3. The genomic evolution of PRV variants

Nowadays, many European and American countries have declared themselves free of PRV infection, and China has effectively controlled PR through Bartha K61 vaccination. Since 2011, outbreaks of PRV infections have occurred on several pig farms in eastern and northern China, and more worryingly, these affected pigs have already been vaccinated against PRV (Yu et al., 2014; Hu D. et al., 2015; Wang Y. et al., 2015). Before 2011, there is only one serotype of PRV, although different PRV isolates differ in their biological characteristics. He et al. constructed the maximum likelihood tree based on the full-length sequence of PRV and showed that current PRV isolates could be divided into two different branches termed as genotype 1 and genotype 2 (He et al., 2018). Construction of the Maximum clade credibility tree using the gC gene confirmed that PRV was divided into clade 1 and 2, while showing that the two clades had been circulating independently for centuries. Maximum likelihood trees were constructed based on gB and gD, respectively, with PRV divided into two clades, and unsurprisingly Bartha K61 and the post-2011 variants were assigned to different clades (He et al., 2018). Interestingly, after 2011 the Chinese PRV isolates were mainly genotype 2, which partly explains why the Bartha K61 vaccine (genotype 1) could not provide complete protection against the novel PRV variants. The gB, gC, and gD are the major antigenic genes and are susceptible to mutation and therefore are regularly using in PRV phylogenetic analysis (Zhai et al., 2019). Independent phylogenetic analysis of antigen genes makes it more readily to understand the relationship between vaccines and different virulent strains. The study demonstrated that the mutations of gC and gD of PRV variants plays the crucial role to escape the immunity induced by Bartha K61 (Ren et al., 2020).

Natural mutations and recombinants promote variation in the diversity of PRV strains. Zhai et al. collected PRV samples from pig farms in eastern China from 2017–2019. Based on the gC gene, these PRVs belong to clade 2. Sequencing analysis of the major PRV glycoproteins gB, gC, gD, and gE showed maximum amino acid sequence divergences of 4.0, 0.7, 0.3, and 1.1%, respectively, within the isolates. Compared with Bartha K61 vaccine strain these isolates have mutations at several protein sites, especially S41G, R162H, and S345L changes on gC could alter receptor-binding and/or glycosylation, which could lead to the emergence of variant PRV (Zhai et al., 2019). Natural recombination accelerates the evolution of the viral genome, and bioinformatics analysis indicates that interclade and intraclade recombination frequently occurs in the PRV genomes (Hu et al., 2021). Tan et al. found a novel PRV strain of HN2019 derived from the recombination of classical PRV (such as Ea) and HB-98 vaccine strains (Tan et al., 2022). Hu et al. prediction of recombination with Bartha K61 for all Chinese isolates yield 23 recombination events (Hu et al., 2021). Bo et al. found that the Bartha strain provided part of the genes for the recombinant JSY13 variant (Bo et al., 2021). The early PRV strain SC is a recombinant strain between a Chinese endemic strain and a Bartha vaccine-like strain (Ye et al., 2016). These reports suggest that recombination plays a critical role in the evolution of PRV in China. Bartha K61 was introduced into China nearly half a century ago, and long-term immunization has greatly contributed to the mutation and recombination of PRV. Therefore, surveillance and evolution analysis of PRV are important for the prevention and control for PR diseases while this information could provide key information for the development and use of PRV vaccines.

## 4. Live PRV vaccine

**Figure 2:**
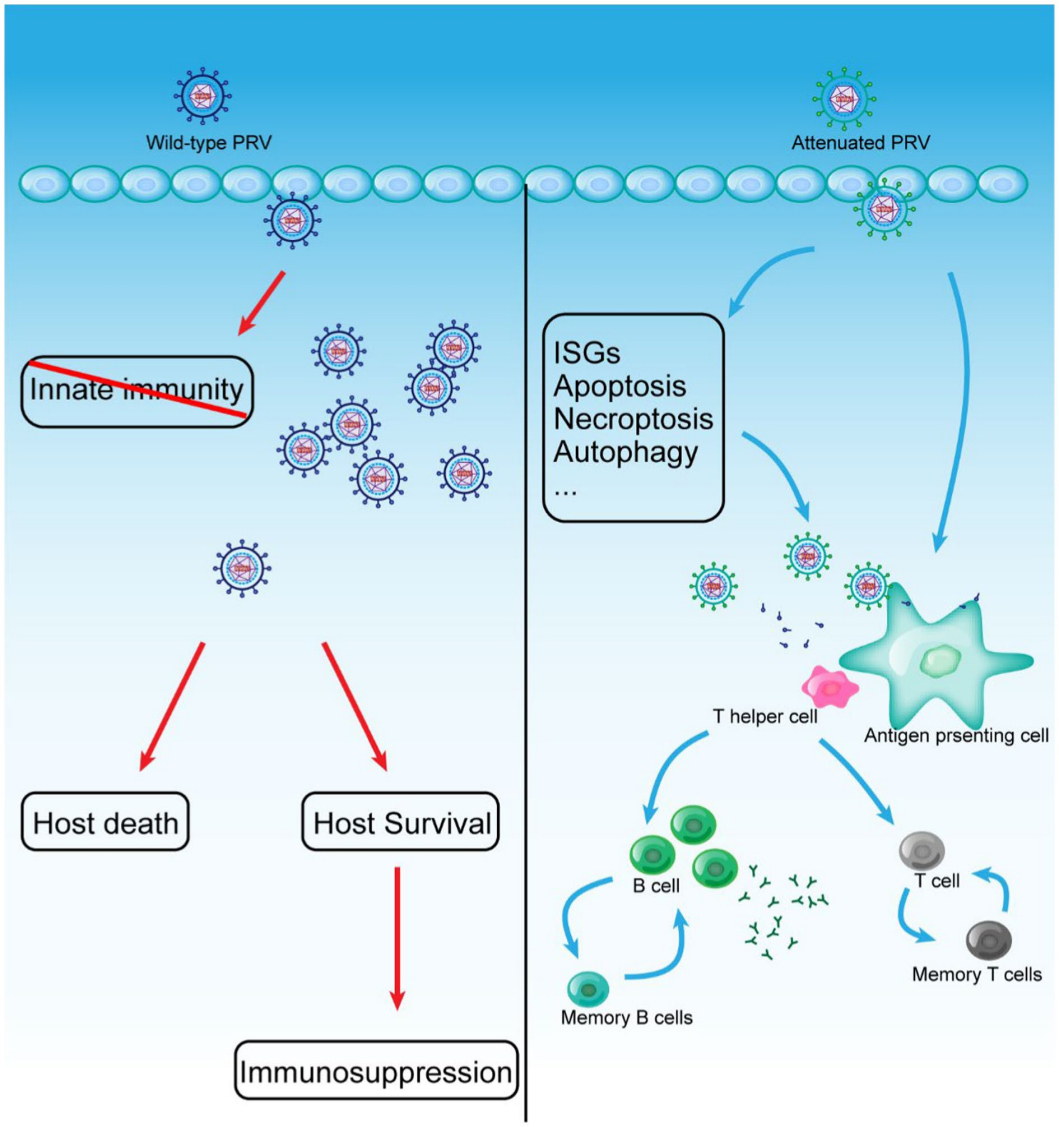
WT PRV and Live attenuated vaccines induce the establishment of adaptive immunity *in vivo*.

Vaccination is one of the most effective strategies against infectious diseases (Jordan et al., 2022; Oduwole et al., 2022). The success of the PRV eradication campaign is mainly attributed to the application of the excellent PRV vaccine (McFerran and Dow, 1970; Sun et al., 2016; Freuling et al., 2017). However, traditional vaccination is not a once-and-for-all solution, and the human struggle against infectious diseases is ongoing. The epidemic of PRV variants in China prompts us to focus on developing new and more effective vaccines to stop the PRV variants pandemic. Attenuated vaccines retain most of the natural structure of the pathogen. Still, they have reduced replication capacity and virulence *in vivo*, and it is now generally accepted that attenuated vaccines provide better and more complete immune protection, especially T-cell-mediated immunity (Figure 2; Van Rooij et al., 2000). Currently, live attenuated vaccines are the most wildly applied and developed. This section summarized the current progress and key issues in the development of live attenuated PRV vaccines and live PRV vector-based vaccines, and provided insights into the development of novel live vaccines.

### 4.1. Live attenuated vaccine

Most PRV vaccines are live attenuated vaccines based on partial genetic deletions. The US region genes US3, US7 and, US8 of PRV are the major virulent genes and are considered non-core genes that are not important for PRV replication (Pomeranz et al., 2005; Wang J. et al., 2020; Zhao et al., 2020). US2 is conserved in most herpesviruses and indirectly regulates MAPK activity by regulating the localization of ERK (Kang et al., 2013). Another study has shown that deletion of the PRV US2 gene increases viral titers (Lyu et al., 2018). US9 is a membrane protein that performs a vital role in the cis-neural transport of herpesviruses, including PRV (Kramer et al., 2012). UL23 encodes a nonstructural protein PK associated with PRV virulence and is barely immunogenic (Yan et al., 2021; Tan et al., 2022). Currently these genes are often used as PRV attenuators.

The first live attenuated PRV Bartha K61 strain is one of the best modified live vaccines (Delva et al., 2020). The Bartha K61 vaccine strain was serially passaged *in vitro* and obtained attenuated properties (Bartha, 1961). Later, the researchers found many deletions in the US region of Bartha K61, including complete deletion of the US8 and US9 genes and partial deletion of US7 and US2 (Mettenleiter et al., 1985; Petrovskis et al., 1986). With advances in PRV genetics and molecular biology, the role of PRV genes is being unrecovered, and more gene-deleted PRV vaccines are being developed by a various technologies (Tomishima and Enquist, 2001; Pomeranz et al., 2005; Bo and Li, 2022). Genetic engineering techniques such as homologous recombination, and bacterial artificial chromosome (BAC) and CRISPR/Cas9 gene editing system allow rapid construction of gene-deleted strains for as live attenuated vaccine candidates. This mainly includes single-gene deletion, double-gene deletion, triple-gene deletion, quadruple-gene deletion, and quintuple-gene deletion vaccines (Table 2).

**Table 2:** Overview of live attenuated PRV vaccine.

Gene-Deleted Vaccines	Vaccine Strains	Deleted Gene(s)	Growth characteristics compared with parents	Target animals	References
Single-gene deletion	TJ	gE	Significantly lower growth kinetics	Piglet	Wang C. H. et al. (2014)
Double-gene deletion	JS-2012	gE/gI	Similar growth kinetics, significantly reduced plaque size	Piglet and pregnant sow	Tong et al. (2016)
	XJ	gI/gE	Similar growth kinetics	Piglet	Yin et al. (2017)
	AH02LA	TK/gE	Significantly lower growth kinetics	Piglet and mice	Wang et al. (2018)
Triple-gene deletion	AH02LA	TK/PK/gE	Similar growth kinetics compared to the PRV^ΔTK&gE-AH02^	Piglet and mice	Xu et al. (2022b)
	NY	gE/gI/TK	Similar growth kinetics	Mice	Zhao et al. (2020)
	XJ5	gI/gE/TK	Unclear	Piglet	Wang J. et al. (2020)
	SMX	gI/gE/TK	Growth kinetics were slow in the first 8 h after inoculation and similar thereafter	Piglet, growing pig and sheep	Hu R. M. et al. (2015)
	GL	US7/US8/UL23	Similar growth kinetics	Dog	Yin et al. (2020)
	QYY2012	gE/TK/US3	Slightly decreased growth kinetics	Mice	Deng et al. (2022)
Quintuple-gene deletion	GL	US7/US8/UL23/US3	Significantly lower growth kinetics	Dog	Yin et al. (2020)
	GDFS	gI/gE/US9/US2	Unclear	Piglet	Sun L. et al. (2022)
Quadruple-gene deletion	HN1201	TK/gE/gI/11 k/28 k	Similar growth kinetics, significantly reduced plaque size	Piglet	Yan et al. (2021)
	GX	TK/gI/gE/US9/US2	Slightly reduced plaque size and slightly decreased growth kinetics	Mice and piglet	Li L. et al. (2022)

Despite the tremendous significance of live attenuated vaccines for PR prevention and control (Freuling et al., 2017; Mettenleiter, 2020), some researchers have questioned the safety of live attenuated vaccines in susceptible animals (Kong et al., 2013; Lin et al., 2019; Yin et al., 2020). The attenuated vaccine strain rPRVTJ-delgE/gI was avirulent in pigs but lethal in sheep (Cong et al., 2016), suggesting that deletion of gE and gI alone may not be sufficient for attenuation. Another group constructed a TK/gE double gene deletion vaccine based on the PRV AH02LA strain, which resulted in the death of PRV antibody-free 1-day-old piglets (Wang et al., 2018). Deletion of the PK gene based on PRV^ΔTK&gE-AH02^ resulted in further attenuation of the PRV virus, and no clinical signs were observed after inoculation of newborn piglets without PRV antibodies (Xu et al., 2022b). A recent study found that further deletion of the US3 gene based on the gE/TK deletion strain had better immunogenicity in mice with higher levels of neutralizing antibodies, cytokines, and tissue-resident memory T cells (TRM; Deng et al., 2022). Remarkable is the US3-encoded PK protein, which is extensively involved in the immune escape of PRV as summarized in the section 1.2. Therefore, the US3 gene could be a promising target for live attenuated vaccine design and development.

More and more mutant-based attenuated PRV vaccines have been developing but there are still several issues that need to further consideration. Firstly, the attenuators are still limited to single or multiple deletions of US2, US3, US7, US8, US9, and UL23, novel attenuation-related genes are need to further exploited next step; secondly, little attention has been paid to the viral growth characteristics after multi-gene deletion, some gene deletion may reduce the virus titers to affect the production of live attenuated vaccines; finally, the safety of the vaccine in animals other than pigs needs to be further studied, especially in the nonhuman primates. To control the new PRV variants, updating the vaccine strain to match the circulating strains is most critical. Therefore, it is essential to continue to monitor changes of the field strains and to construct new vaccine candidates accordingly. Our general concept is that the safety of vaccines is positively correlated with the number of virulence-associated gene deletions (Mikolajczak et al., 2014; Cong et al., 2016; Yin et al., 2020), but more gene deletions have a greater impact on vaccine strain immunogenicity and production. Weakening viral immune evasion is a strategy for constructing attenuated vaccines. Previous studies revealed that US3, US8, UL13, UL16, UL24, UL42, UL50, and EP0 help PRV to evade host innate immunity, therefore these genes have the potential to construct an attenuated vaccine. The introduction of immune escape genes could lead to reduce the virulence of PRV and improve its safety. For example, Wang et al. constructed EP0 deletion mutant of PRV is more sensitive to IFN-α-mediated antiviral immunity (Wang M. et al., 2022). Another team constructed a UL50-deleted mutant of PRV similarly sensitive to IFN-α-mediated viral inhibition (Zhang et al., 2017). Despite the lack of animal studies to validate the ability of these genes to reduce virulence, the introduction of PRV immune escape genes into current live attenuated vaccines is a promising strategy to generate novel PRV live vaccines.

### 4.2. Live attenuated recombinant PRV vaccine against multiple pathogens

Pigs are threatened by at least 40 pathogens, which include 16 viruses, 15 bacteria, 8 parasites, and 1 protozoan (VanderWaal and Deen, 2018). In recent years, pig infectious diseases such as African swine fever (ASF), foot-and-mouth disease (FMD), PR, porcine reproductive and respiratory syndrome (PRRS), porcine parvovirus (PPV) and influenza, etc., have been widely documented (Dixon et al., 2020; Sehl and Teifke, 2020; Kanankege et al., 2022; Kim et al., 2022; Li C. et al., 2022; Sun Y. et al., 2022; Zhang X. et al., 2022). Vaccination with the appropriate vaccine for each potential epidemic outbreak is the most effective method, but there are many mixed infections in field (Sun et al., 2016; Saade et al., 2020). The correct vaccination of different vaccines several times in a short culture cycle is difficult to achieve. The insertion and expression of heterogeneous antigenic genes in the viral genome leading to additional immune protection or enhanced immune effect is an exciting research topic (DiNapoli et al., 2007; Wang et al., 2019; Ma et al., 2022). PRV has a large genome, and the deletion of some genes, as described earlier, renders it attenuated without significantly affecting its growth ability and immunogenicity. Several PRV-based vector vaccines have been developed. E.g., co-expressing the PrM-E of Japanese encephalitis virus (JEVs; Qian et al., 2015), E2 of classical swine fever virus (CSFVs; Lei et al., 2016; Tong et al., 2020), FMDV P12A and 3C (Li et al., 2008; Zhang et al., 2011), PPV VP2 (Chen et al., 2011), Porcine deltacoronavirus (PDCoV) spike (S; Huang et al., 2022), PRRSV GP5 and M (Zhao J. et al., 2022), CP204L (p30), CP530R (pp62), E183L (p54), B646L (p72), and EP402R (CD2v) genes of ASFV (Chen L. et al., 2022) and SARS-CoV-2 S or N (Li R. et al., 2022). These multivalent vaccines have demonstrated preventive effects against many diseases, but further research is needed on their safety and efficacy in pig. These studies showed that gene-deleted attenuated PRV virus could be a promising vector to develop multivalent vaccines against two or more pathogens.

Molecular adjuvants enhance vaccine efficacy by modulating the immune system through multiple mechanisms (Batista-Duharte et al., 2018, 2022; Weiss et al., 2022). Designing suitable molecular adjuvants to construct a live recombinant vaccine based on gene-deficient PRV is a promising direction. Zheng et al. constructed recombinant PRV expressing PPV VP2 protein and porcine IL-6, significantly increasing PPV-specific lymphocyte proliferation (Zheng et al., 2020). Fms-related tyrosine kinase 3 ligand (Flt3L) insertion into TK and gE deficient PRV to construct recombinant HNX-TK^−^/gE^−^-Flt3L significantly enhances maturation and activation of dendritic cells (DCs; Yao et al., 2021). Granulocyte-macrophage colony-stimulating factor (GM-CSF) is a cytokine produced by immune cells that activates macrophages and promotes dendritic cell development (Bukreyev et al., 2001; Hamilton, 2008). Its potential value as a vaccine adjuvant is gradually being explored, and Luo et al. constructed a strain of GM-CSF using HDR-CRISPR/Cas9 technology (Luo et al., 2022). It exhibited higher protective effects than live attenuated PRV not expressing GM-CSF, including higher levels of specific neutralization antibodies, and cellular immune response. Mycobacterium tuberculosis heat shock protein 70 c-terminal (HSP70c) is a potent TLR2 agonist (Bulut et al., 2005). Park et al. co-inoculation of pigs with PRRSV attenuated vaccine and HSP70c showed a significant increase in PRRSV-specific IFN-γ-SCs and cytokine levels (Park et al., 2021). PRV is also recognized by TLR2, and it is interesting to insert HSP70c within the genome to enhance live attenuated PRV vaccines. In conclusion, PRV has a high tolerance for insertion of exogenous genes and different molecular adjuvants for the construction of recombinant PRV vaccines.

## 5. Conclusion

PRV is still one of the significant infectious agents in pigs. Also, the continuous occurrence of human infections with PRV has increased the level of risk of PRV. PRV uses multiple strategies to evade host innate immunity. Modification or deletion of innate immune escape genes are an promising strategy for the development of novel live attenuated vaccines. Live attenuated vaccine strain is also a good vector to develop the novel multivalent vaccines against two or more pathogens including viruses, bacteria and mycoplasma, especially against the respiratory-related pathogens. In addition, the insertion of suitable molecular adjuvants can greatly improve the efficiency of vaccine protection. Although several PRV vaccines have been applied in clinical, it is urgent to accelerate the development of novel vaccines against the current epidemic of PRV variants and other pathogens.

## Author contributions

ZN and SZ wrote the first draft of the manuscript. HF contributed to conception and design of the review. All authors contributed to the article and approved the submitted version.

## Funding

This research was supported by “Pioneer” and “Leading Goose” R&D Program of Zhejiang under Grant Nos. 2022C02031, 2023C02047, and 2023C02023, by National Natural Science Foundation of China under Grant No. 32172893, by the Fundamental Research Funds of Zhejiang Sci-Tech University under Grant No. 2021Q035, by Zhejiang Provincial Natural Science Foundation of China under Grant Nos. LY22C180002 (For HF) and LQ22C180003 (For LW), by Science Foundation of Zhejiang Sci-Tech University (ZSTU) under Grant No. 20042220-Y and by the Open Fund of the Shaoxing Academy of Biomedicine of Zhejiang Sci-Tech University under Grant No. SXAB202019.

## Conflict of interest

The authors declare that the research was conducted in the absence of any commercial or financial relationships that could be construed as a potential conflict of interest.

## Publisher’s note

All claims expressed in this article are solely those of the authors and do not necessarily represent those of their affiliated organizations, or those of the publisher, the editors and the reviewers. Any product that may be evaluated in this article, or claim that may be made by its manufacturer, is not guaranteed or endorsed by the publisher.
